# Recombinant HcGAPDH Protein Expressed on Probiotic *Bacillus subtilis* Spores Protects Sheep from *Haemonchus contortus* Infection by Inducing both Humoral and Cell-Mediated Responses

**DOI:** 10.1128/mSystems.00239-20

**Published:** 2020-05-12

**Authors:** Yi Yang, Guiheng Zhang, Jie Wu, Xueqiu Chen, Danni Tong, Yimin Yang, Hengzhi Shi, Chaoqun Yao, Lenan Zhuang, Jianbin Wang, Aifang Du

**Affiliations:** a College of Animal Sciences, Zhejiang Provincial Key Laboratory of Preventive Veterinary Medicine, Institute of Preventive Veterinary Medicine, Zhejiang University, Hangzhou, China; b Ross University School of Veterinary Medicine and One Health Center for Zoonoses and Tropical Veterinary Medicine, Ross University School of Veterinary Medicine, Basseterre, St. Kitts, West Indies; c Department of Biochemistry and Molecular Genetics, RNA Bioscience Initiative, University of Colorado School of Medicine, Aurora, Colorado, USA; University of Illinois at Chicago

**Keywords:** *Bacillus subtilis*, *Haemonchus contortu*s, spore coat, microbiota, recombinant live vaccine

## Abstract

Probiotic *Bacillales* are effective in controlling pathogens. Live probiotic bacteria improve the composition of the gastrointestinal microbiota, leading to a reduction in pathogen colonization. However, it remains largely unknown how probiotics regulate the host’s immunologic responses and protect the host from parasitic infection. In this study, we addressed whether *Bacillales* were effective against *Haemonchus contortus*, a parasitic nematode that infects small ruminants worldwide. Using 16S rRNA sequencing, we found that *Bacillales* were largely depleted in the abomasal microbiota of sheep infected with *H. contortus*. We constructed a recombinant Bacillus subtilis named rBS*^CotB-HcG^* that express the glyceraldehyde-3-phosphate dehydrogenase of *H. contortus* (HcGAPDH) on its spore surface using the Bacillus subtilis spore coat protein B (CotB) as a carrier. Mice receiving rBS*^CotB-HcG^* orally showed strong Th1-dominated immune responses. More importantly, sheep administered BS*^CotB-HcG^ per os* showed increasing proliferation of the peripheral blood mononucleates, elevated anti-HcGAPDH IgG in sera, and higher anti-HcGAPDH sIgA in the intestinal mucus than the control sheep. The average weight gain of *H. contortus*-infected sheep treated with rBS*^CotB-HcG^* (Hc+rBS*^CotB-HcG^*) was 48.73% greater than that of unvaccinated sheep. Furthermore, these Hc+rBS*^CotB-HcG^* sheep had fewer eggs per gram of feces by 84.1% and adult worms by 71.5%. They also demonstrated greatly lessened abomasal damage by *H. contortus* with an abundance of probiotic species in the abomasal microbiota. Collectively, our data unequivocally demonstrate the protective roles of CotB-HcGAPDH-expressing B. subtilis spores in against *H. contortus* infection and showed great potential of using probiotic-based strategy in controlling parasitic nematodes of socioeconomic importance in general.

**IMPORTANCE** Initial analyses of the abomasal microbiota of sheep using 16S rRNA sequencing suggested that probiotic bacteria played a protective role in against *H. contortus* infection. A recombinant Bacillus subtilis expressing a fusion protein CotB-HcGAPDH on its spore’s surface induced strong Th1 immune response in a murine model. The same probiotic recombinant, upon only one oral application, protected sheep against *H. contortus* infection by reducing egg shedding and decreasing adult worm loads of the parasite and increasing body weight gain of infected sheep. Both Th1 and Th2 immune responses were evident in these immunized sheep.

## Abstract (precis)

Initial analyses of the abomasal microbiota of sheep using 16S rRNA sequencing suggested that probiotic bacteria played a protective role in against *H. contortus* infection. A recombinant Bacillus subtilis expressing a fusion protein CotB-HcGAPDH on its spore’s surface induced strong Th1 immune response in a murine model. The same probiotic recombinant, upon only one oral application, protected sheep against *H. contortus* infection by reducing egg shedding and decreasing adult worm loads of the parasite and increasing body weight gain of infected sheep. Both Th1 and Th2 immune responses were evident in these immunized sheep.

## INTRODUCTION

*Haemonchus contortus* is one of the most economically important parasites causing haemonchosis in small ruminants around the world (1). The latter may lead to anemia, weakness, and even death in infected hosts (2). To control *H. contortus* infection and minimize economic losses brought upon the ruminant industry by haemonchosis, anthelmintics have been widely used. Consequently, *H. contortus* populations resistant to anthelmintics have emerged and become prevalent in many geographic regions (3). New prevention strategies against haemonchosis are urgently needed.

Probiotics are known to promote human and animal health. In particular, probiotics from food sources reduce intestinal infections by such pathogens as porcine rotavirus (4). Another study showed that Bacillus subtilis inhibited the colonization of Staphylococcus aureus in sheep and goats (5). Further, B. subtilis has been widely used as a vehicle for oral vaccines in animals (6–8). A recent study showed that the Bacillus subtilis spore coat protein C (CotC), a major component of the B. subtilis spore coat, was able to carry Clonorchis sinensis cysteine protease on the bacterial spore surface (9). Recombinant B. subtilis spores expressing a tegumental protein was shown to provide protection against C. sinensis infection in a rat model (10).

*H. contortus* glyceraldehyde-3-phosphate dehydrogenase (HcGAPDH), an important excretory/secretory component of the worm, is a glycolytic enzyme (11, 12). A recombinant *HcGAPDH* DNA vaccine reduces *H. contortus* infection in sheep (13). However, this DNA vaccine has not been put into wide use, likely due to its limited commercial availability (14). Therefore, a more practical and better protection strategy against haemonchosis is needed. The purposes of this study were to develop an oral vaccine using recombinant B. subtilis spores expressing a CotB-HcGAPDH fusion protein, to demonstrate its protective role, and to investigate its underlying mechanisms.

## RESULTS

### The relative abundance of *Bacillales* negatively correlated with *H. contortus* infection.

**FIG 1:**
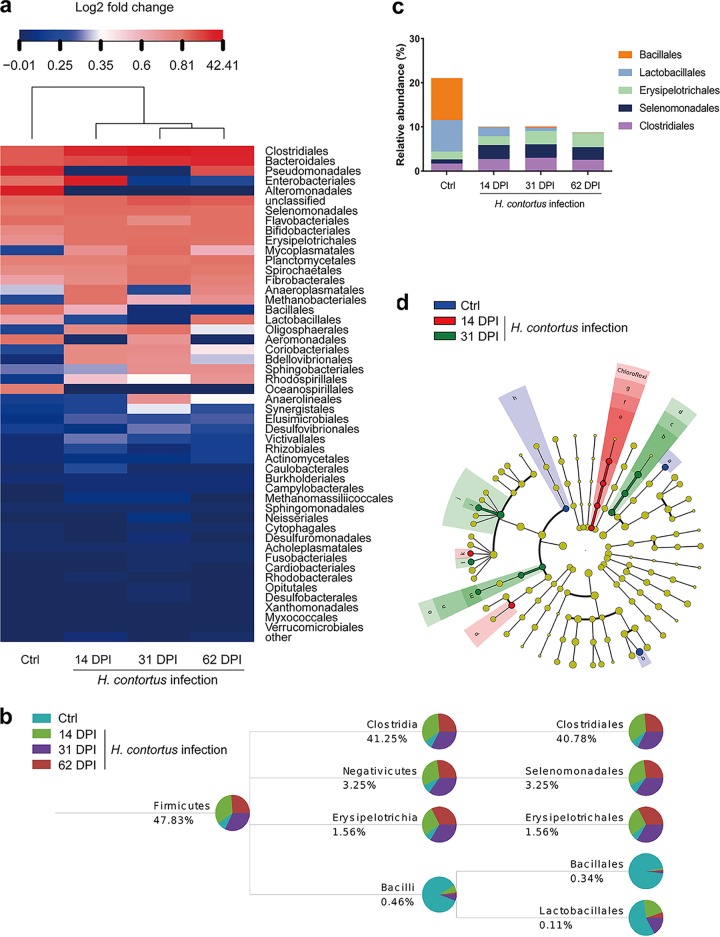
The relative abundance of *Bacillales* is related to *H. contortus* infection in sheep. (a) Heatmap of relative abundance of abomasal bacteria. Color breaks in the heatmap are adjusted to show the relative abundance at <0.3% (blue shades), 0.3 to 0.4% (white shades), and >0.4% (red shades). DPI, day postinfection. (b) Community taxonomic system composition analysis of *Firmicutes*. The relative abundance of abomasal bacteria in each sample is shown by a colored pie chart. (c) Taxonomic composition of *Firmicutes*. The proportion of different color blocks indicates the relative abundance of different species. (d) Taxonomic cladogram obtained by linear effect size (LEfSe) analysis of 16S sequences within groups. Different colors represent different groups, and differently colored nodes in the branches represent groups of microorganisms that play an important role in the corresponding group of colors (a, *Myroides*; b, *Sphingobacteriaceae*; c, *Sphingobacteriales*; d, *Sphingobacteria*; e, *Anaerolineaceae*; f, *Anaerolineales*; g, *Anaerolineae*; h, *Bacilli*; i, *Lachnospiracea_incertae_sedis*; j, *Lachnospiraceae*; k, *Pseudoflavonifractor*; l, *Ruminococcus*; m, *Bulleidia*; n, *Erysipelotrichales*; o, *Erysipelotrichia*; p, *Veillonellaceae*; q, *Psychrobacter*).

To investigate the effect of microbiota on *H. contortus* infection, we analyzed abomasal microbiota of *H. contortus*-infected sheep using 16S rRNA (rRNA) sequencing. In the control sheep without *H. contortus* infection, the abomasal microbiota were dominated by the following bacterial classes: *Alteromonadales* (35.5%), *Pseudomonadales* (29.5%), *Bacteroidales* (10.4%), *Clostridiales* (9.8%), *Flavobacteriales* (3.4%), *Enterobacteriales* (1.9%), *Bacillales* (1.3%), and *Aeromonadales* (1.0%) (Fig. 1a). *H. contortus* infection induced dramatic changes in microbial abundance, including those of *Alteromonadales*, *Pseudomonadales*, *Sphingobacteriales*, *Enterobacteriales*, *Bacillales*, and *Coriobacteriales*, compared to the uninfected group (Fig. 1a). Of particular interest were the *Bacillales* bacteria that had probiotic effects in relation to *H. contortus* infection. The relative abundance of *Bacillales* was significantly reduced upon *H. contortus* infection (Fig. 1b and c) (*P* < 0.005). It was further shown by linear effect size (LEfSe) analysis of the 16S rRNA sequences that *Bacillales* was the main contributor as a probiotic in the abomasal microbiota to protect sheep from *H. contortus* infection (Fig. 1d). Together, these data demonstrated that sheep with *H. contortus* infection have significant reductions in *Bacillales* levels in the abomasum, suggesting a potential protective role of these probiotic bacteria in against nematodes and possibly other pathogenic infection.

### Expression of CotB-HcGAPDH on the surface did not affect the production and structure of *B. subtilis s*pores.

**FIG 2:**
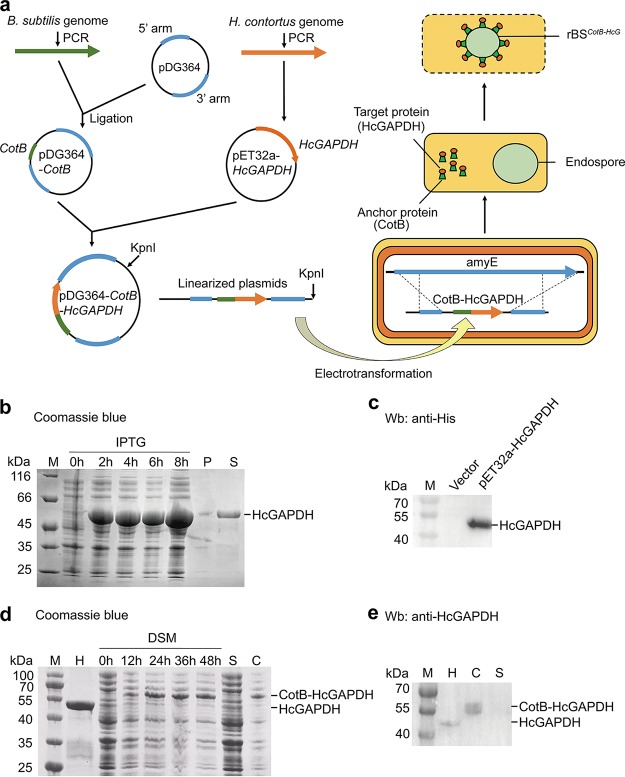
Recombinant B. subtilis spores expressing CotB-HcGAPDH on the surface. (a) Schematic of genetic engineering to generate recombinant spores with CotB-HcGAPDH presenting on the surface (rBS*^CotB-HcG^*). (b) Coomassie blue staining of SDS-PAGE gel showing the recombinant HcGAPDH protein. M, protein marker; HcGAPDH, recombinant GAPDH from *H. contortus*; P, pellet; S, supernatant; IPTG, isopropyl β-d-thiogalactoside. (c) Western blot of recombinant HcGAPDH with anti-His antibody. Vector, empty pET32a control. (d) Coomassie blue staining of the CotB-HcGAPDH fusion protein in B. subtilis. H, purified HcGAPDH protein; S, supernatant; C, spore coat from rBS*^CotB-HcG^;* DSM, Difco sporulation medium. (e) Western blot of CotB-HcGAPDH fusion protein probed with polyclonal anti-HcGAPDH rabbit antibody.

We then generated recombinant spores expressing CotB and the HcGAPDH protein (CotB-HcGAPDH or CotB-HcG) as a fusion protein on their surfaces (Fig. 2a). This was achieved in two steps. First, the full-length cDNA of *HcGAPDH* was cloned into the pET32a vector (pET32a-HcGAPDH), followed by the expression and purification of the recombinant HcGAPDH protein (Fig. 2b and c). The purified protein was then used to generate polyclonal antibodies in rabbit. Second, the *CotB* and *HcGAPDH* genes were fused in order and cloned into the pDG364 vector (pDG364-*CotB*-*HcGAPDH*). The fusion protein CotB-HcGAPDH (CotB-HcG) was expressed in B. subtilis spores (rBS*^CotB-HcG^*) (Fig. 2d and e).

**FIG 3:**
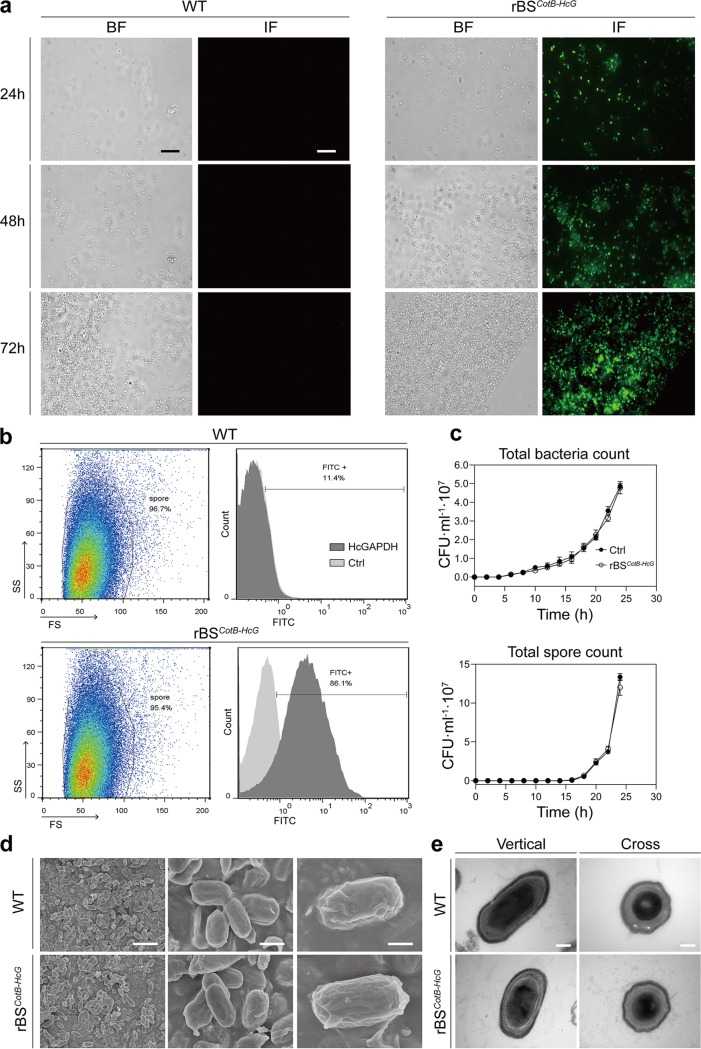
Expression of CotB-HcGAPDH fusion protein did not affect the structure and production of B. subtilis spores. (a) Immunofluorescence (IF) of CotB-HcGAPDH expressed on the surfaces of wild-type (WT) and rBS*^CotB-HcG^* spores. The different time points (24, 48, and 72 h) after spore induction by DSM are indicated. BF, bright field; IF, immunofluorescence. Scale bar, 1 μm. (b) Flow cytometry analysis of CotB-HcGAPDH expression on the surfaces of WT and rBS*^CotB-HcG^* spores. FS, forward scatter; SS, side scatter. (c) Production and germination analysis of WT and rBS*^CotB-HcG^* spores. (d) Representative images of WT and rBS*^CotB-HcG^* spores obtained by scanning electron microscopy. Scale bars: 10 μm (left) and 1 μm (middle and right). (e) Representative images of WT and rBS*^CotB-HcG^* spores obtained by transmission electron microscopy. Scale bars: 200 nm (left) and 100 nm (right).

To verify that the recombinant fusion protein CotB-HcGAPDH was expressed on the surfaces of B. subtilis spores, immunofluorescence was performed using rabbit polyclonal antibodies to HcGAPDH on the bacterial spores rBS*^CotB-HcG^* that was induced in Difco sporulation medium (DSM). CotB-HcGAPDH in rBS*^CotB-HcG^* started to appear on the spore coat after 24 h of induction and increased steadily from 24 to 72 h (Fig. 3a). Flow cytometry assay further confirmed that 86.01% of the rBS*^CotB-HcG^* spores expressed CotB-HcGAPDH 72 h after induction (Fig. 3b). No differences between wild-type (WT) and rBS*^CotB-HcG^* strains were observed in the production and germination of spores (Fig. 3c). rBS*^CotB-HcG^* spores were further examined to determine whether expression of CotB-HcGAPDH affected spore structure by both scanning electron microscopy and transmission electron microscopy. Again, no change was observed in the coat folds of elliptical spore morphology related to the wild type (Fig. 3d). The exine and intine structures of rBS*^CotB-HcG^* were similar to those of the wild-type strain (Fig. 3e). These results indicate that expression of the CotB-HcGAPDH fusion protein did not change the production and structure of B. subtilis spores.

### Recombinant *B. subtilis* spores expressing CotB-HcGAPDH fusion protein stimulated both humoral and cell-mediated immune responses in mice and sheep.

**FIG 4:**
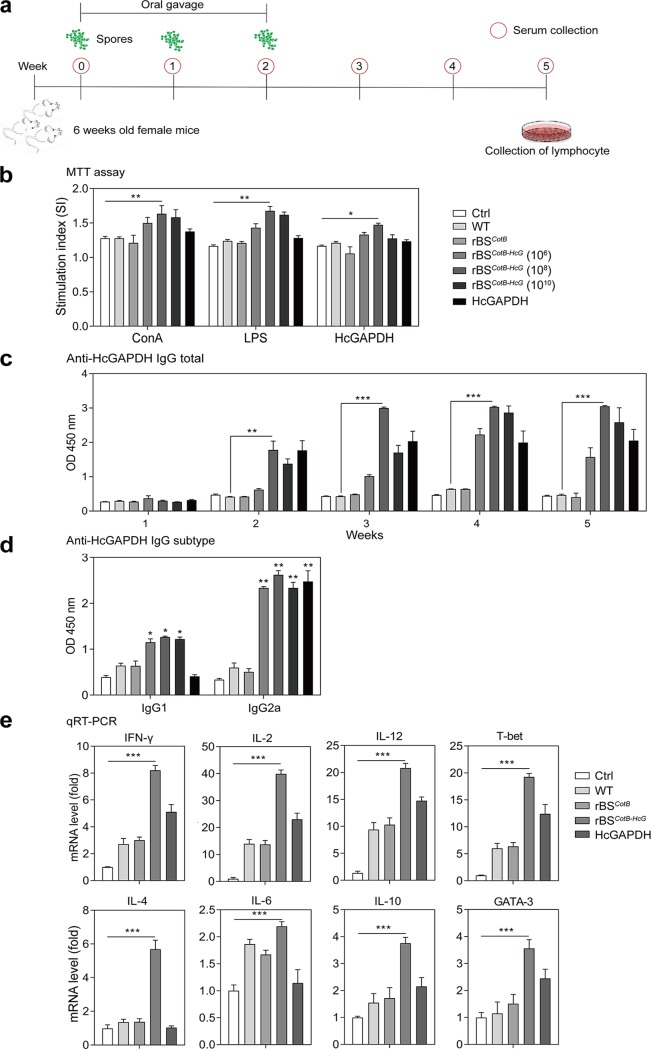
Recombinant B. subtilis spores expressing the CotB-HcGAPDH fusion protein induced both humoral and cell-mediated immune responses in mice. (a) Schematic of the experimental protocol in mice. Six-week-old female mice (*n* = 20 in each group) administered PBS (Ctrl); WT, rBS*^CotB^*, rBS*^CotB-HcG^* spores; or purified HcGAPDH protein at the indicated dosages. Serum was collected at the indicated time points. (b) Proliferation of splenic lymphocytes of mice (*n* = 6 in each group) measured by an MTT assay. (c) Anti-HcGAPDH IgG levels in sera of mice (*n* = 6 in each group) at different time points. (d) IgG1 and IgG2a levels in murine sera at week 5 (*n* = 6 in each group). (e) mRNA levels of cytokine and transcription factor genes in splenic lymphocytes from mice (*n* = 6 in each group) measured by qRT-PCR. The dosage of rBS*^CotB-HcG^* was 10^10^ CFU. *, *P *≤* *0.05; **, *P *≤* *0.01; ***, *P *≤* *0.005. All data are presented as means ± the SEM. Three technical replicates from a single experiment were used.

To test whether the recombinant B. subtilis spores have positive probiotic effects on promoting immune responses, mice were orally administered phosphate-buffered saline (PBS; Ctrl), WT strain, rBS*^CotB^* or rBS*^CotB-HcG^* spores, and purified HcGAPDH protein, respectively (Fig. 4a). Lymphocytes prepared from the murine spleens were cultured and stimulated with concanavalin A (ConA), lipopolysaccharide (LPS), or the purified HcGAPDH protein to determine the specific cell-mediated immune responses. Higher levels of lymphocyte proliferation were observed in rBS*^CotB-HcG^*-treated mice than in control mice receiving ConA or LPS stimulation (*P* < 0.01) (Fig. 4b). Similar results were obtained when the purified HcGAPDH protein was used as a stimulator (*P* < 0.05). Further, the anti-HcGAPDH immunoglobulin G (IgG) levels in these murine sera were measured. The highest antibody level was detected in mice given rBS*^CotB-HcG^* (*P* < 0.005) at week 3 (Fig. 4c). No anti-HcGAPDH antibody was detected in mice receiving PBS or the WT or rBS*^CotB^* strain (*P* > 0.05). The anti-HcGAPDH IgG2a level was 2.07 times higher than that of anti-HcGAPDH IgG1 (*P* < 0.005), indicating a Th1-dominated T cell immune response (Fig. 4d). We next determined the levels of the secretory IgA (sIgA) to HcGAPDH in the intestinal epithelial cells and plasma cells. These were significantly higher in rBS*^CotB-HcG^* mice than in controls (*P* < 0.01) (see Fig. S1 in the supplemental material). Genes representing Th1 activation, such as gamma interferon (IFN-γ), interleukin-2 (IL-2), IL-12, and T-bet, and those of Th2 activation, such as IL-4, IL-6, IL-10, and GATA-3, in the splenic lymphocytes were significantly induced by rBS*^CotB-HcG^* administration in comparison to controls (Fig. 4e), suggesting that rBS*^CotB-HcG^* stimulated mixed Th1/Th2 immune responses. Collectively, these data indicate that B. subtilis spores expressing the CotB-HcGAPDH fusion protein activated both humoral and cell-mediated immune responses in mice.

10.1128/mSystems.00239-20.1 FIG S1 The CotB-HcGAPDH fusion protein expressing recombinant B. subtilis spores induced both humoral and cell-mediated immune responses in sheep. (a) Anti-HcGAPDH sIgA levels in intestinal mucous samples of mice (*n* = 6 in each group). (b) Anti-HcGAPDH sIgA levels in intestinal mucous samples of sheep (*n* = 6 in each group). Download FIG S1, TIF file, 1.4 MB. Copyright © 2020 Yang et al. 2020 Yang et al. This content is distributed under the terms of the Creative Commons Attribution 4.0 International license.

**FIG 5:**
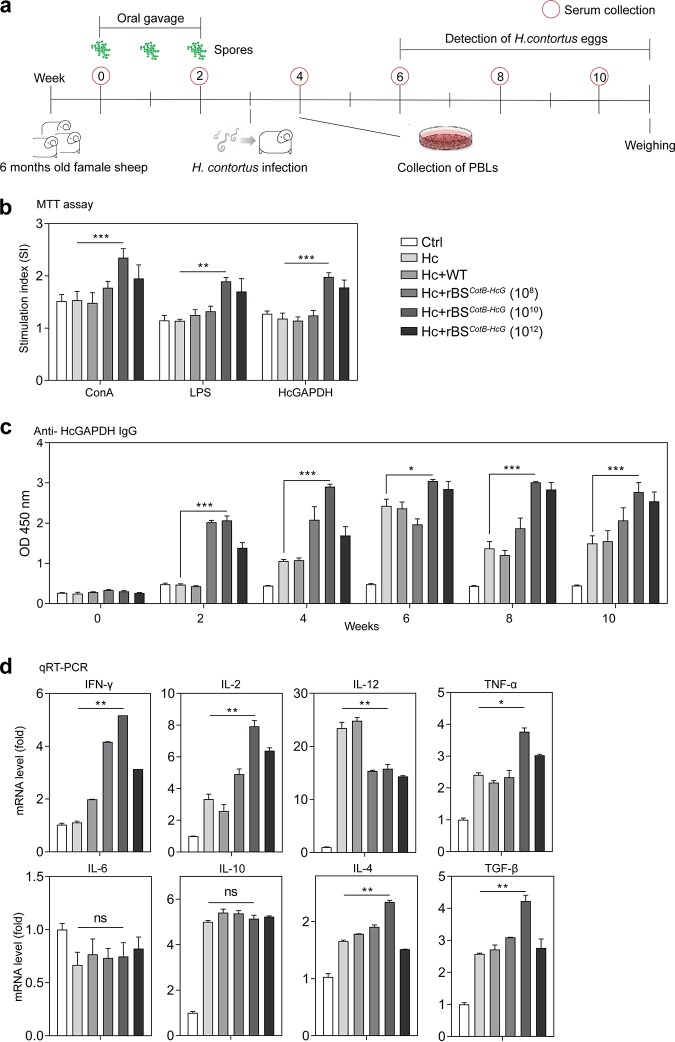
The CotB-HcGAPDH fusion protein expressing recombinant B. subtilis spores stimulated both humoral and cell-mediated immune responses in sheep. (a) Schematic of the experimental protocol in sheep. Six-month-old female sheep were fed by gavage with PBS (Ctrl) or with WT or rBS*^CotB-HcG^* spores at the indicated dosages during the first 3 weeks, followed by *H. contortus* infection (*n* = 6 per group). Serum samples were collected at the indicated time points. (b) Proliferation of PBLs as measured by an MTT assay at week 4 (*n* = 6 in each group). (c) Anti-HcGAPDH IgG levels in sera of sheep (*n* = 6 in each group). (d) The mRNA levels of cytokine and transcription factor genes in peripheral blood lymphocytes (PBLs) of sheep (*n* = 6 in each group) were measured by qRT-PCR at week 4. *, *P* ≤ 0.05; **, *P* ≤ 0.01, ***, *P* ≤ 0.005. All data are presented as means ± the SEM. Three technical replicates from a single experiment were used.

We next investigated the immune responses stimulated by the rBS*^CotB-HcG^* spores in sheep, one of the natural hosts of *H. contortus*. An *in vivo* experiment was carried out *per os* through gavage with PBS (control [Ctrl]), *H. contortus* infection (Hc), wild-type (WT) strain, or rBS*^CotB-HcG^* spores, followed by *H. contortus* infection, designated Ctrl, Hc, Hc+WT, and Hc+rBS*^CotB-HcG^*, respectively (Fig. 5a). Peripheral blood lymphocytes (PBLs) from sheep were isolated on day 7 after infection, which is the time required for the infective L3 (iL3) of *H. contortus* to develop to the blood-sucking L4 stage in the abomasum. These cells were cultured and stimulated with ConA, LPS, or purified HcGAPDH protein. Consistent with the murine results, the proliferation of PBLs from the sheep receiving Hc+rBS*^CotB-HcG^* in the presence of ConA or LPS was significantly greater than that from the Hc group (*P* < 0.005) (Fig. 5b). The purified HcGAPDH protein also stimulated significant proliferation of PBLs from these sheep in comparison to control sheep (*P* < 0.005). The administration of rBS*^CotB-HcG^* induced anti-HcGAPDH IgG production (*P* < 0.005, compared to the Ctrl) at week 2, and the IgG level plateaued at week 4 until week 8 (Fig. 5c). Meanwhile, anti-HcGAPDH IgG was not detectable in the control sheep. Further, anti-HcGAPDH sIgA levels were significantly higher in the intestinal mucus of Hc+rBS*^CotB-HcG^* sheep than in that of the Hc sheep (*P* < 0.01) (Fig. S1). It was also found that genes representing Th1 activation (IFN-γ, IL-2, IL-12, and tumor necrosis factor alpha [TNF-α]) and those of Th2 activation (IL-4 and transforming growth factor β [TGF-β]) in PBLs of Hc+rBS*^CotB-HcG^* sheep were highly activated (Fig. 5d), even though the expression of IL-6 and IL-10 did not change (*P* > 0.05). Collectively, these data show that rBS*^CotB-HcG^* stimulated strong humoral and cell-mediated immune responses in both mice and sheep.

### CotB-HcGAPDH recombinant *B. subtilis* spores promoted the relative abundance of probiotic bacilli in abomasal microbiota in sheep.

**FIG 6:**
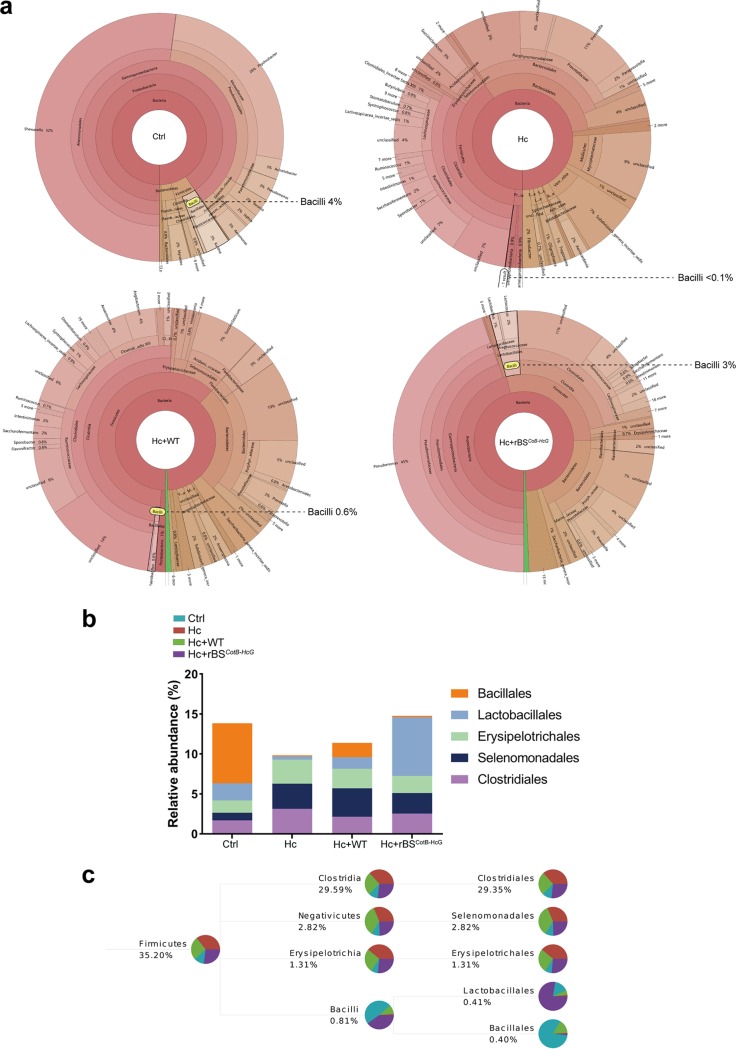
The CotB-HcGAPDH fusion protein expressing recombinant B. subtilis spores promoted relative abundance of probiotic bacilli in the abomasal microbiota in sheep. (a) Hierarchical analyses depicted in the form of a Krona plot. 6-month-old female sheep orally gavaged with PBS (Ctrl) or with WT or rBS*^CotB-HcG^* spores in 10^12^ CFU/animal, followed by *H. contortus* infection (*n* = 3 in each group in all data). (b) Community taxonomic system composition analysis at the class level in sheep. (c) Taxonomic composition of *Firmicutes*. The proportions of different color blocks indicate the relative abundances of different species.

To investigate whether administration of rBS*^CotB-HcG^* affected abomasal microbiota of sheep in concomitant with *H. contortus* infection, 16S rRNA gene was sequenced from the abomasal mucosal samples collected from the sheep of different treatment groups. Bacilli accounted for <0.1% in the abomasal microbiota of sheep with *H. contortus* infection compared to 4% of the controls (Fig. 6a), which was consistent with our earlier findings (Fig. 1). Bacilli from Hc+rBS*^CotB-HcG^* sheep accounted for 3%, indicating that administration of rBS*^CotB-HcG^* could restore bacilli depleted by *H. contortus* infection (Fig. 6a). Community taxonomic system composition analysis of *Firmicutes* indicated that administration of rBS*^CotB-HcG^* increased the relative abundance of *Lactobacillales* (Fig. 6b). Specifically, *Lactobacillales* accounted for 19.6, 0.1, 3.9, and 76.8% of *Firmicutes* in Ctrl, Hc, Hc+WT, and Hc+rBS*^CotB-HcG^* animals, respectively (Fig. 6c). These results indicate that administration of rBS*^CotB-HcG^* spores improved the composition of the microbiota by increasing the ratio of probiotic species in the abomasa of sheep infected with *H. contortus*.

### CotB-HcGAPDH recombinant *B. subtilis* spores protected sheep from *H. contortus* infection.

**FIG 7:**
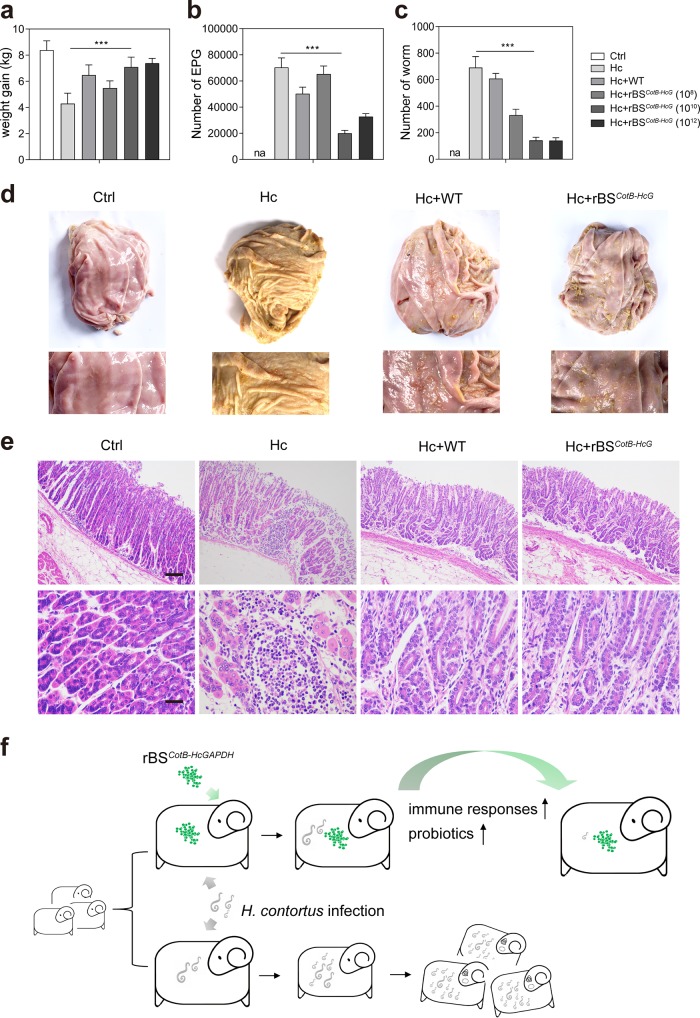
The CotB-HcGAPDH recombinant B. subtilis spores protected sheep from *H. contortus* infection. (a) Average weight gain of sheep (*n* = 6 in each group). All data in this figure were collected from sheep according to the protocol shown in Fig. 5a. (b) Number of eggs per gram (EPG) of feces from sheep (*n* = 6 in each group). (c) Number of worms from abomasum of sheep (*n* = 6 in each group). (d) Representative pictures of abomasum in sheep (upper panel). The lower, magnified images show the *H. contortus* in abomasum in sheep (lower panel). (e) HE staining of abomasa in sheep. All values in panels a, b, and c are presented as means ± the SEM. *, *P* ≤ 0.05; **, *P* ≤ 0.01, ***, *P* ≤ 0.005. (f) Schematic of the protective effect of the CotB-HcGAPDH recombinant B. subtilis spores on *H. contortus* infection.

To study the protective effect of rBS*^CotB-HcG^* on sheep against *H. contortus* infection, we measured the body weights and parasite loads of infected sheep. The average weight of the *H. contortus* infected sheep was only 43.6% of that of the noninfected control sheep, while sheep receiving rBS*^CotB-HcG^* at 10^10^ or 10^12^ CFU/animal, followed by *H. contortus* infection, had body weights close to the uninfected controls. The WT B. subtilis spores also showed some degree of protection against body weight loss caused by *H. contortus* infection. In this case, 27.7% more body weight was recorded in the sheep receiving the WT B. subtilis spores than to the infected sheep without it (Fig. 7a). Next, parasite load by egg per gram feces (EPG) and adult worm counting was determined. For sheep given 10^10^ CFU of rBS*^CotB-HcG^*/animal, followed by *H. contortus* infection, the EPG levels dropped by 71.5% (Fig. 7b), and the worm load dropped by an astonishing 84.1% compared to sheep infected with *H. contortus* alone (Fig. 7c and Table 1). We also evaluated the infection by examining the abomasum. The abomasal surfaces of infected sheep were covered with worms and tracks of parasite crawling. The numbers of worms and parasite crawling tracks in the Hc+rBS*^CotB-HcG^* sheep decreased compared to Hc group (Fig. 7d). Furthermore, the abomasal mucosas of infected sheep showed intensive infiltration by mononucleates in comparison to the uninfected sheep. No apparent infiltration was observed in Hc+rBS*^CotB-HcG^* sheep (Fig. 7e). These data indicate that rBS*^CotB-HcG^* offer effective protection of sheep from *H. contortus* infection by improving abomasal microbiota (Fig. 7f).

**TABLE 1:** Worm reduction rates, EPG reduction rates, and body weight loss in sheep receiving different treatments^*a*^

Treatment	Worm count			Egg count			Body wt gain (kg)			
	Mean	SD	Worm reduction (%)^*b*^	Mean	SD	EPG reduction (%)^*c*^	Mean	SD	wt loss^*d*^	wt loss recovery (%)^*e*^
PBS	0	0	NA	0	0	NA	8.09	1.24	NA	
Hc	690.8	205.0		70,350.0	17,753.96		4.56	1.29	3.53	
Hc+WT (10^12^)	609.8	93.9	11.8	50,300.0	12,145.45	28.5	6.36	1.23	1.73	50.99**
Hc+rBS*^CotB-HcG^* (10^8^)	332.5	106.5	51.9**	65,366.7	14,908.21	7.1	5.39	1.10	2.70	23.51
Hc+rBS*^CotB-HcG^* (10^10^)	109.7	33.6	84.1***	20,050.0	5,528.38	71.5***	6.59	1.89	1.50	57.51***
Hc+rBS*^CotB-HcG^* (10^12^)	140.8	52.5	79.6***	32,766.7	5,481.85	53.4**	6.28	1.71	1.81	48.73**

a EPG, eggs per gram of feces; NA, not appicable. *, *P* ≤ 0.05; **, *P* ≤ 0.01; ***, *P* ≤ 0.005.

b Calculated as (Hc-individual treatment)/Hc × 100%.

c Calculated as (Hc-individual treatment)/Hc × 100%.

d PBS-Hc or individual treatment.

e Calculated as (Hc-individual treatment)/Hc × 100%.

## DISCUSSION

The goals of the present study were to evaluate protective capacity of HcGAPDH engineered on the B. subtilis spore surface in sheep against infection by *H. contortus* and to elucidate the immunologic mechanisms of its protection. A recombinant B. subtilis strain rBS*^CotB-HcG^* was developed by expression of *H. contortus HcGAPDH* fused to *CotB* on the spore coat. Such recombination and heterologous expression did not yield detectable changes in the production and structure of the spores. rBS*^CotB-HcG^* regulated the abomasal microbiota in favoring the host sheep, especially when they were infected by *H. contortus* with perturbed microbiota in the abomasum. rBS*^CotB-HcG^* induced Th1-dominated immune responses in a mouse model. The same mechanism may offer effective protection for sheep from *H. contortus* infection and also alleviated damage triggered by parasitic infections.

The method of vaccine delivery greatly affects the recipient’s immune responses (15). Oral vaccination has great potential for field use since large amounts of particulate materials can be delivered with low risk of adverse effects (16). More importantly, the probiotic-based strategy of vaccination can alleviate the use of anthelmintics, thus reducing anthelmintic residues in food and milk and at the same time minimizing anthelmintic resistance of parasites. Antibodies in the mucus are the first line of host’s defense against various pathogens, including parasites, invading the mucosa (17–19) by inhibiting the motility and adherence of the pathogens (20). Both sIgA and IFN-γ have powerful bactericidal effects in early infection (21). However, whether oral administration is the best immunization strategy for *H. contortus* remains to be verified. Our data indicate that B. subtilis recombinant spores resisted the harsh conditions in the gastrointestinal tract and oral immunization with recombinant B. subtilis spores activated a strong mucosal immune response in the intestinal mucosa (Fig. S1).

It has been shown that antigen delivery via bacterial spores produces a Th1-biased cellular response, as demonstrated by high levels of IgG2a (22). IL-12 is a key cytokine that induces Th1-type immune response (15). The transcription factor T-bet is a major regulator of Th1 cell polarization (23). Significant upregulation of IL-12 and T-bet gene expression induced by rBS*^CotB-HcG^* indicated that B. subtilis spores mainly elicited Th1-type immune responses in a murine model. Interestingly, our results also indicate that the administration of rBS*^CotB-HcG^* in sheep induced Th2-type immune responses besides Th1, as shown by the upregulation of cytokine IL-4 and TGF-β, implying that there may be mixed Th1/Th2 immune responses in sheep (24, 25). A plausible explanation for this is that such mixed immune responses are jointly activated by spores and HcGAPDH antigenic protein. Alternatively, rBS*^CotB-HcG^* by proteolytic cleavage releases soluble antigens, including HcGAPDH, following their uptake by antigen-presenting cells (APCs), which leads to presentation to a major histocompatibility complex class II (MHC-II)-restricted manner for the generation of Th2-type immune responses (26). IL-4 is a signal cytokine for the Th2 response and is mainly responsible for the IgE isotype switch (27). The immunosuppressive cytokine IL-10 is responsible for the inhibition of Th2 immune responses (28, 29). In mice, a slight upregulation of cytokines (IL-4 and IL-10) and Th2-type transcription factors (GATA-3) was found, suggesting that rBS*^CotB-HcG^* could induce Th1/Th2 mixed immune responses. TGF-β manipulates various immune activities differentially in various cell types and potentially regulates a wide range of biological processes. In sheep, the mRNA levels of both TGF-β and IL-2 of lymphocytes in the peripheral blood were increased. Some cytokines, particularly IFN-γ and TGF-β, have previously been proved to induce the upregulation of both MHC-I and MHC-II gene expression in different immune cells (30), which then stimulates the production of antibodies and immune responses against parasitic pathogens (30). Therefore, the upregulation of TGF-β gene expression in sheep receiving rBS*^CotB-HcG^* suggested that spores presenting HcGAPDH protein activate the host immune responses against parasitic infections by stimulating both MHC-I and MHC-II antigen-presenting pathways. Our results are consistent with an early study using different recombinant spores (31).

Here, we have shown an example of a live recombinant probiotic bacterium expressing a subunit vaccine that protects recipients against *H. contortus* infection. *H. contortus* infection leads to significant decrease in the abundance of *Bacillales* in the abomasal microbiota. A correlation of *H. contortus* infection with specific changes at the species or genus level of bacteria was not established in the present study. One plausible explanation is that sequencing-based approach is intended to detect taxonomic shifts at a level higher than the genus/species level. This is consistent with an earlier report (5). *Bacillus* spp. are widely used as probiotics in the livestock industry lately. Currently, a few European Union-approved products are available in the market. The most notable one is BioPlus2B from Christian Hansen (32). The probiotic B. subtilis emerges favorably as a vaccine carrier is because of its protective effects against a wide spectrum of pathogens (16, 33–35). In addition, its spores possess adjuvant property due to a combination of antigens and the spore surface (36, 37). Many *Bacillus* species are safe for sheep and can be used in sheep feeds (38). The present study unequivocally demonstrated that a recombinant B. subtilis, rBS*^CotB-HcG^*, offers great protection against *H. contortus* infection in sheep, a natural host of this devastating nematode for the sheep industry.

**FIG 8:**
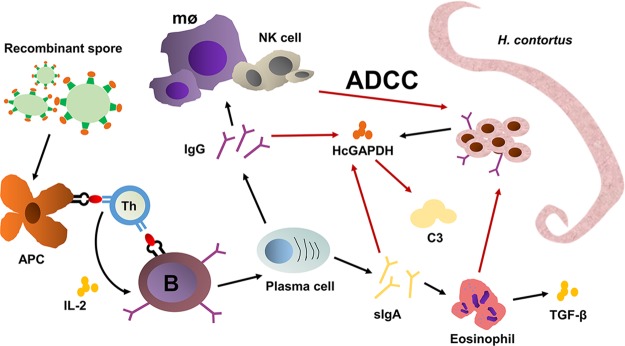
Hypothetical scheme for recombinant spores expressing the CotB-HcGAPDH fusion protein in protecting sheep from *H. contortus* infection. Antigen-presenting cells (APC) refer to a type of immune cell capable of ingesting and processing antigens and then presenting the treated antigens to T and B lymphocytes. Th, helper T cell could help B cell produce antibodies. B, B lymphocyte. mø, macrophages. ADCC, antibody-dependent cell-mediated cytotoxicity.

Currently, the mechanism that this recombinant organism vaccine works is not well understood. Our working hypothesis (Fig. 8) is that rBS*^CotB-HcG^* activates T helper lymphocytes by APCs and stimulates increasing the release of IL-2 that synergistically activates B lymphocytes to transform them into plasma cells. The latter generates anti-HcGAPDH IgG antibodies. The spores also stimulate intestinal epithelial cells and plasma cells to produce anti-HcGAPDH sIgA, which facilitates the proliferation of eosinophils and the upregulation of TGF-β, resulting in parasite killing and clearance. HcGAPDH plays a key role in the inhibition of host complement activation. Antibody neutralization of HcGAPDH removes this inhibitor, leading to complement activation. Further, live *H. contortus* worms release HcGAPDH that is involved in evasion of the host immune system. Anti-HcGAPDH IgG and sIgA hence block the immune evasion of *H. contortus.*

## MATERIALS AND METHODS

### Ethics approval.

Animal use was approved by Zhejiang University Experimental Animals Ethics Committee (permit ZJU20160239). All animals were cared for in accordance with guidelines for care and use of laboratory animals set by the same committee.

### Parasite and animals.

*H. contortus* Zhejiang strain was kept in the Veterinary Parasitology Laboratory, Zhejiang University, and maintained by serial passage in helminth-free sheep. Infective L3 larvae (iL3s) were obtained by incubation of eggs for 14 days at 28°C.

Six-week-old female BALB/c mice were purchased from the Zhejiang Academy of Medical Science (Hangzhou, China). The mice were raised in a sterilized room with the temperature set at 26 to 27°C, with a 12-h daily light cycle, and fed sterilized food and water. Six-month-old female sheep were purchased from the Miemieyang Animal Husbandry Co., Ltd. (Huzhou, China). All sheep were housed indoor with a natural daily light cycle. They were provided with hay and whole corn as food three times a day, with the same quantity for each group, and water *ad libitum*. Rabbits were purchased from Zhejiang Academy of Medical Science (Hangzhou, China) at the age of 6 months.

### Sheep abomasal microbiota.

Six-month-old female sheep were each orally infected with 5,000 *H. contortus* iL3s suspended in 1 ml of PBS. They were euthanized at 14, 31, or 62 days postinfection (dpi). The control sheep received 1 ml of plain PBS and were sacrificed at 62 dpi. These sheep were housed in separated areas of the same building within the Miemieyang Animal Husbandry Co., Ltd., to minimize cross-contamination. A portion (10 ml) of abomasum fluids was collected from each sheep within 20 min of euthanasia. The abomasal fluids were centrifuged at 5,000 × *g* for 5 min at 4°C. The supernatants were centrifuged at 12,000 × *g* for 10 min at 4°C, and the pellet of the second centrifugation was analyzed for 16S rRNA sequences by using an Illumina MiSeq platform (Sangon Biotech, China) to obtain the abomasal microbiota of each sheep. Raw sequences have been deposited in the Sequence Read Archive database under project number SRP217048. Python v1.2.2 was used to analyze both heatmap and community taxonomic system composition. LEfSe v1.1.0 was used to analyze taxonomic cladogram. Krona v2.6.1 was used for hierarchical analyses.

### Plasmid construction.

The 1,023-bp coding sequence (CDS) of HcGAPDH was amplified from the total cDNA of *H. contortus* by PCR using primers previously described (10). PCR products were sequenced in both directions (BioSune, China) after being cloned to the pET-32a vector (TaKaRa, China), resulting in the pET32a-HcGAPDH plasmid.

To generate a recombinant spore carrying the CotB-HcGAPDH, genomic DNA of B. subtilis strain 168 was used as the template to amplify the *CotB* gene of 1,088 bp, which included the promoter of 263 bp and a partial N-terminal CDS of 825 bp. The PCR primers are listed in Table S1. PCR products were cloned into the pMD 18-T vector (TaKaRa, China). The *CotB* and *HcGAPDH* genes were fused in order and cloned into the pDG364 vector (pDG364-*CotB*-*HcGAPDH*). The *CotB*-*HcGAPDH* was amplified by PCR with primers (Table S1). The fused *CotB-HcGAPDH* was subcloned into E. coli-B. subtilis shuttle vector pDG364 (Miaolingbio, China), resulting in pDG364-CotB-HcGAPDH plasmid. The control plasmid pDG364-*CotB* was constructed by cloning the *CotB* gene to the pDG364 vector. All cloned DNAs were confirmed without mutations by sequencing (BioSune, China).

10.1128/mSystems.00239-20.2 TABLE S1 Primers used in the present study. Download Table S1, DOCX file, 0.01 MB. Copyright © 2020 Yang et al. 2020 Yang et al. This content is distributed under the terms of the Creative Commons Attribution 4.0 International license.

### Expression of recombinant proteins.

The recombinant vector pET32a-HcGAPDH was transformed into E. coli. BL21 bacteria. The transformants were induced with 0.5 mM IPTG (isopropyl-β-d-thiogalactopyranoside) after being cultured until the optical density at 600 nm (OD_600_) reached 0.6 at 37°C. The cells were then broken apart by sonication after centrifugation at 8,000 × *g* for 10 min and resuspension in a buffer (0.01% digitonin, 10 mM PIPES [pH 6.8], 300 mM sucrose, 100 mM NaCl, 3 mM MgCl_2_, and 5 mM EDTA) with proteinase inhibitors. Soluble His-tagged HcGAPDH was purified from bacterial lysate using a HisTrap column (GE Healthcare Life Sciences). HcGAPDH purity was checked by SDS-PAGE gel stained with Coomassie blue. The anti-HcGAPDH rabbit polyclonal antibodies (rAb) were prepared according to the previous method (16). The purified protein HcGAPDH and anti-HcGAPDH rAb were stored at −80°C.

The linearized pDG364-CotB-HcGAPDH plasmid was transformed into the genome of B. subtilis strain 168 by electroporation (39) at the location of amylase E gene by homologous recombination. B. subtilis spores were generated in 4 liters of DSM with 25 μg/ml chloramphenicol at 37°C for sporulation of the recombinant B. subtilis rBS*^CotB-HcG^* or rBS*^CotB^*, as previously described (22). The spores were purified by treatment with 4 mg/ml lysozyme, followed by washing in 1 M NaCl and 1 M KCl solution with 1 mM phenylmethylsulfonyl fluoride under stringent conditions, as described previously (19). The resultant preparations were then treated at 68°C for 1 h in water to remove any residual sporangial cells. The spores were kept at −80°C at a concentration of 1 × 10^12^ CFU/ml in PBS (pH 7.4) until use.

### SDS-PAGE and Western blotting.

Spores of the recombinant B. subtilis rBS*^CotB-HcG^* transfectant were generated as previously described (22). The spores were harvested and analyzed for the presence of HcGAPDH protein by SDS-PAGE. Further, spore coat proteins were extracted from the spores at 48 h of bacterial incubation in DSM using SDS-DTT extraction buffer (0.5% SDS, 0.1 M dithiothreitol, 0.1 M NaCl) as previously described (39). The extracted proteins were subjected to 12% SDS-PAGE, followed by transfer onto a polyvinylidene fluoride filter (Sigma, Germany). The latter was blocked overnight at 4°C in 5% skimmed milk in PBST (PBS with 0.05% [vol/vol] Tween 20), followed by incubation in anti-HcGAPDH rAb (1:1,000 in PBST) as the primary antibody and horseradish peroxidase-conjugated goat anti-rabbit IgG (1:5,000 in PBST) as the secondary antibody. The signal was detected by using enhanced chemiluminescence (Beyotime Biotechnology, China).

### Immunofluorescence and flow cytometry assay.

For immunofluorescence, 5-ml portions of sporulation cultures at 24, 48, or 72 h of incubation were harvested and processed as previously described (10). Spores were blocked with 5% bovine serum albumin for 2 h at 4°C, followed by incubation with anti-HcGAPDH rAb (1:2,000 in PBST) for 2 h at room temperature. Naive preimmunized rabbit sera (1:2,000 in PBST) were used as a negative control. Fluorescein isothiocyanate (FITC)-conjugated goat anti-rabbit IgG (Invitrogen, 1:500 in PBST) was used as the secondary antibody. Spores were observed and photographed under a fluorescence microscope (Olympus BX51, Japan) equipped with an Olympus camera (Olympus Micro DP72, Japan).

For flow cytometry, 10^5^ purified spores were washed three times in PBS and then incubated with anti-HcGAPDH rAb (1:500 in PBST) at 37°C for 2 h. Naive rabbit sera (1:500 in PBST) was used as a negative control. After being washed three times in PBS, the spores were incubated with FITC-conjugated goat anti-rabbit IgG (1:500 in PBST; Invitrogen) at 37°C for 1 h. The spores were finally resuspended in 1 ml of PBS after three washes, and a minimum of 10^4^ spores were examined by using an FC500 MPL flow cytometer (Beckman Coulter). Expression of the CotB-HcGAPDH fusion protein was analyzed using FlowJo software (TreeStar).

### Analysis of the production and structure of the recombinant spores.

The purified WT and recombinant rBS*^CotB-HcG^* spores were collected and fixed in 3% glutaraldehyde overnight at 4°C, followed by dehydration in gradient ethanol at 50, 70, 90, and 100%. After subsequent critical point drying and sputter coating, the samples were processed and photographed under a SU-70 scanning electron microscope (Hitachi, Japan). For transmission electron microscopy, the spores were fixed in glutaraldehyde overnight at 4°C, followed by incubation in 4% osmium tetroxide for 2 h. Next, they were dehydrated in gradient ethanol (50, 70, 90, and 100%) and embedded in epoxy resin, and the ultrathin sections were mounted on a 230-mesh copper mesh stained with 1% uranyl acetate-lead citrate. The spores were observed and photographed under a H-9500 transmission electron microscope (Hitachi, Japan).

To investigate whether the production of spores of the recombinant rBS*^CotB-HcG^* was different from that of the wild type, both strains were individually inoculated in 1 liter of DSM and then cultured at 37°C with constant shaking at 140 r/min. The numbers of viable bacteria and spores were then quantified as previously described (19).

### Animal experiments.

Six-week-old female BALB/c mice were administered 100 μl of PBS (Ctrl), WT spores at 1 × 10^10^ CFU (WT), rBS*^CotB^* spores at 1 × 10^10^ CFU (rBS*^CotB^*), or rBS*^CotB-HcG^* spores at 10^6^, 10^8^, or 10^10^ CFU (rBS*^CotB-HcG^*) per mouse by oral gavage. There were 20 mice in each group. The mice in all groups, including Ctrl mice, were originally immunized with three doses applied daily on three consecutive days, followed by two boosts at a 1-week interval. Each boost was administered the same way as the original immunization. Mice in the HcGAPDH group were each subcutaneously immunized with 200 μg of purified HcGAPDH emulsified in the complete Freund’s adjuvant, followed by two boosts with 100 μg of HcGAPDH emulsified in the incomplete Freund’s adjuvant 1 week apart. All mice were euthanized at week 5 after the last boosting. Lymphocytes were isolated from spleens and cultured for the extraction of total RNA.

Thirty-six 6-month-old female sheep were assigned to six groups with six animals per group; each group was assigned control or individual treatment. Each group of sheep was kept in a separated pen housed indoors in the same building. All sheep were provided with food three times a day, with same quantity for each group, and water of the same source *ad libitum.* The entire experiment was conducted in Huzhou, China, between September and November 2018. The temperature was ranged from 32 to 25°C (maximum) to 15 to 30°C (minimum) for this period. Each sheep was administered 1 ml of PBS as the control (Ctrl), the WT spores (WT) at 1 × 10^12^ CFU per sheep (Hc+WT), or rBS*^CotB-HcG^* spores at 1 × 10^8^, 10^10^, or 10^12^ CFU per sheep (Hc+rBS*^CotB-HcG^*) by oral gavage, depending upon its assignment. All sheep except the Ctrl animals were individually challenged with 5,000 *H. contortus* iL3s 1 week later. Serum samples were collected from the jugular vein of each animal every 2 weeks. All sheep were sacrificed at 2 months postinfection with *H. contortus* iL3s. PBLs were isolated at 5 dpi using a sheep peripheral blood lymphocyte separation kit (Sangon Biotech, China). Body weight was obtained using a floor scale (Ohaus D52P150RTL2ZH; Shanghai, China) with a sensitivity of 0.01 kg. The body weight gain of each sheep was recorded as the difference in body weight (kg) between weeks 11 and 0. Weight loss recovery of vaccine-immunized sheep versus unimmunized controls (Hc) was calculated as follows: (body weight loss of Hc group-body weight loss of individual treatment)/body weight loss of Hc group × 100%. The eggs per gram (EPG) value was assayed at 14 dpi according to the modified McMaster method (29). The EPG reduction rate was calculated by: (average EPG in control – average EPG in treatment)/average EPG in control × 100%. The numbers of *H. contortus* adult worms from the abomasum in sheep were counted as described previously (40, 41) after euthanasia at week 11. The worm reduction rate was calculated as follows: (average worm count in control – average worm count in treatment)/average worm count in control × 100%.

### Lymphocyte proliferation assay.

As described previously (42), murine splenic lymphocytes were stimulated with LPS (5 μg/ml; Sigma, Germany), ConA (10 μg/ml; Sigma, Germany), or purified HcGAPDH protein (15 μg/ml). The cells were evaluated for proliferation by using an MTT assay kit (Sangon Biotech, China) according to the manufacturer’s instructions. Experiments with sheep PBLs were performed as described for the murine lymphocytes except for using LPS, ConA, and purified HcGAPDH protein at 10, 15, and 25 μg/ml, respectively.

### qRT-PCR assay.

Total RNA was extracted from PBLs. The cDNA synthesized by using a qPCR-RT kit (Toyobo, Japan) was subjected to quantitative real-time PCR (qPCR) to measure the mRNA levels of cytokines and transcription factors with SYBR green PCR master mix (Applied Biosystems) on a StepOnePlus real-time PCR system (Applied Biosystems). The primers specific for the mouse or sheep *TGF-β*, *IFN-γ*, *IL-2*, *IL-12*, *IL-4*, *IL-6*, *IL-10*, *T-bet*, and *GATA-3* genes are listed in Table S1 in the supplemental material.

### Determination of antibody levels by ELISA.

Serum was collected weekly from each mouse after administration of the spores. The intestinal mucus was collected at the week 5 as previously described (26). The levels of anti-HcGAPDH IgG, sIgA, IgG1, and IgG2a were measured by an enzyme-linked immunosorbent assay (ELISA). Briefly, ELISA plates (Bethyl) were coated with 50 μl of purified HcGAPDH dissolved in coating buffer (0.05 M carbonate-bicarbonate [pH 9.6]) at a concentration of 1,000 ng/ml, followed by incubation in 5% skimmed milk in the coating buffer for 18 h at room temperature. After three washes in PBST, the plates were incubated at 37°C for 2 h in 1:400-diluted serum or mucus in PBST. Subsequently, HRP-conjugated goat anti-mouse IgG (1:5,000 dilutions; Abcam, UK), goat anti-mouse IgA (1:5,000 dilutions; Abcam), or goat anti-mouse IgG1 or IgG2a (1:1,000 dilutions; Abcam) was used as the suitable secondary antibody. After 1 h of incubation, the plates were washed again, and 100 μl of the substrate TMB (3,3′,5,5′-tetramethylbenzidine; BD Biosciences) was added. The reaction was stopped by adding 50 μl of 2 M H_2_SO_4_ after 5 min of incubation in dark, and the plates were read three times at 450 nm in a microplate ELISA reader (Bio-Rad, Japan). Negative-control wells incubated with naive sera were included in each plate. The results are expressed as the average of three OD_450_ values. Anti-HcGAPDH IgG and sIgA levels in ovine serum and mucus, respectively, were similarly analyzed by ELISA. In this case, the secondary antibodies used were HRP-conjugated rabbit anti-sheep IgG and rabbit anti-sheep IgA (1:5,000 dilutions; Abcam), respectively.

### HE staining.

Abomasal sections (5-μm thick) were prepared from formalin-fixed and paraffin-embedded tissue blocks and subjected to hematoxylin and eosin (HE) staining as described previously (30). The samples were then examined under a microscope (Zeiss, Germany).

### Analysis of abomasal microbiota of sheep.

The relative abundances of the abomasal microbiota in sheep from the the Ctrl, Hc, Hc+WT, and Hc+rBS*^CotB-HcG^* groups were analyzed by 16S rRNA gene sequencing. Both sampling and sequencing were processed according to a protocol previously described (43).

### Statistical analysis.

Results are presented as means ± the standard errors of the mean (SEM). One-way analysis of variance was performed. A *P* value of ≤0.05 was considered statistically significant.

### Data availability.

All data supporting the findings of this study are available either within the article or in the supplemental material. Raw abomasal microbiota sequences have been deposited in the Sequence Read Archive (SRA) database under project number SRP217048 (the authors could not make this SRA record available at the time of this paper’s publication due to circumstances related to the COVID-19 pandemic, but it will be made accessible as soon as possible after publication).
