# Supplementary figures and images for: Recombinant HcGAPDH Protein Expressed on Probiotic Bacillus subtilis Spores Protects Sheep from Haemonchus contortus Infection by Inducing both Humoral and Cell-Mediated Responses
Source: mSystems. 2020 May 12;5(3):e00239-20. doi: 10.1128/mSystems.00239-20 (PMC7219552; doi:10.1128/mSystems.00239-20)

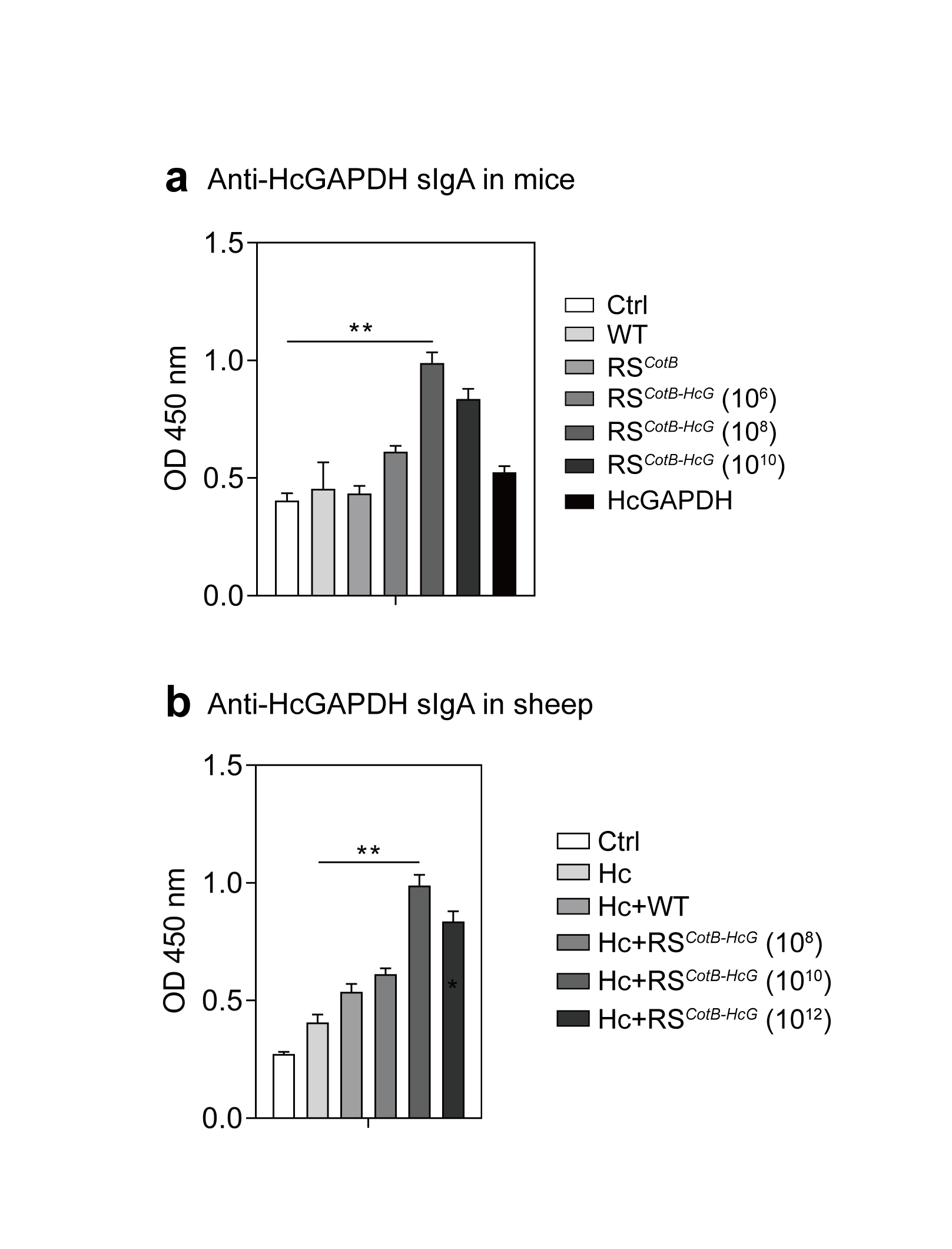

Supplement: FIG S1 [file mSystems.00239-20-sf001.tif]
