# Supplementary material for: Recombinant HcGAPDH Protein Expressed on Probiotic Bacillus subtilis Spores Protects Sheep from Haemonchus contortus Infection by Inducing both Humoral and Cell-Mediated Responses
Source: mSystems. 2020 May 12;5(3):e00239-20. doi: 10.1128/mSystems.00239-20 (PMC7219552; doi:10.1128/mSystems.00239-20)
Supplement: TABLE S1 [file mSystems.00239-20-st001.docx]

**Table S1. Primers used in the current study**

| **Primer ID** | **Primer sequence 5′–3′** |
| --- | --- |
| *CotB-*F  CotB-R | CG**GGATCC**CAGCAAGAGGAGAATGAAATATC  CC**AAGCTT**AAATTTACGTTTCCAGTGATAG |
| *Hc-GAPDH-*F | CC**AAGCTT**ATGGTAAAACCAAAGGTTGG |
| *Hc-GAPDH-*R | CG**GAATTC**TTAGGCCTTGCTTGCAATGTAG |
| S-β-actin R | GCGTAGAGGTCTTTGCGGATGT |
| S-IL-2 F  S-IL-2 R  S-IL-4 F  S-IL-4 R  S-IL-6 F  S-IL-6 R  S-IL-10-F  S-IL-10-R  S-IL-12-F  S-IL-12-R  S-IFN-γ F  S-IFN-γ R  S-TGF-β F  S-TGF-β R  S-TNF-a F  S-TNF-a R  M-β- actin F  M-β- actin R  M-IL-2 F  M-IL-2 R  M-IL-12 F  M-IL-12 R  M-TFN-g F  M-TFN-g R  M-IL-4 F  M-IL-4 R  M-IL-6 F  M-IL-6 R  M-IL-10 F  M-IL-10 R  M-T-bet F  M-T-bet R  M-GATA-3 F  M-GATA-3 R | AACGCTACAGAATTGAAACATC  GTTTCAGATCCCTGTAGTTCCA  TGTTCTGTGAATGAAGCCAAGACGAGTA  ACCCTCATAATAGTCTTTAGCCTTTCCA  AAACGAGTGGGTAAAGAACGCAAAG  GAGGAGGGAATGCCCAGGAACTA  GAGGTGATGCCACAGGCTGAGAA  CTCCACCGCCTTGCTCTTGTTTT  CAGCAGAGGCTCCTCTGAC  GTCTGGTTTGATGATGTCCCTG  TAATGCAAGTAACCCAGATGTA  GCGTAGAGGTCTTTGCGGATGT  GGCAGGTCATCACCATCGGCAAT  GCCGACGTGACAGTAGAGGTAATAGAG  CCAGAGGGAAGAGCAGTCC  GGCTACAACGTGGGCTACC  CATCCGTAAAGACCTCTATGCCAAC  ATGGAGCCACCGATCCACA  CCCAAGCAGGCCACAGAATTGAAA  AGTCAAATCCAGAACATGCCGCAG  GGAAGCACGGCAGCAGAATAAAT  AACTTGAGGGAGAAGTAGGAATGG  TCTTGAAAGACAATCAGGCCATCA  GAATCAGCAGCGACTCCTTTTCC  CAAACGTCCTCACAGCAACG  CTTGGACTCATTCATGGTGC  ACAACCACGGCCTTCCCTACTT  CACGATTTCCCAGAGAACATGTG  GCTCTTACTGACTGGCATGAG  CGCAGCTCTAGGAGCATGTG  GATCATCACTAAGCAAGGACGGC  AGACCACATCCACAAACATCCTG  AGTCCTCATCTCTTCACCTTCC  GGCACTCTTTCTCATCTTGCCT |

*The underlined and highlighted are restriction sites.*
